# Focal loss of the paranodal domain protein Neurofascin155 in the internal capsule impairs cortically induced muscle activity in vivo

**DOI:** 10.1186/s13041-020-00698-y

**Published:** 2020-11-23

**Authors:** Kazuo Kunisawa, Nobuhiko Hatanaka, Takeshi Shimizu, Kenta Kobayashi, Yasuyuki Osanai, Akihiro Mouri, Qian Shi, Manzoor A. Bhat, Atsushi Nambu, Kazuhiro Ikenaka

**Affiliations:** 1 Division of Neurobiology and Bioinformatics, National Institute for Physiological Sciences, Okazaki, 444-8787 Japan; 2 Department of Physiological Sciences, SOKENDAI (The Graduate University for Advanced Studies), Okazaki, 444-8787 Japan; 3 Division of System Neurophysiology, National Institute for Physiological Sciences, 38 Nishigonaka, Myodaiji, Okazaki 444-8585 Japan; 4 Section of Viral Vector Development, National Institute for Physiological Sciences, Okazaki, 444-8585 Japan; 5 Department of Regulatory Science for Evaluation and Development of Pharmaceuticals and Devices, Fujita Health University Graduate School of Health Sciences, Toyoake, 470-1192 Japan; 6 Department of Cellular and Integrative Physiology, School of Medicine, University of Texas Health Science Center, San Antonio, 78229-3900 USA; 7 Department of Neurophysiology and Brain Science, Nagoya City University Graduate School of Medical Sciences, Nagoya, 467-8601 Japan

**Keywords:** Paranodal junction, Multiple sclerosis, Electromyogram, Motor system, Neurofascin155

## Abstract

Paranodal axoglial junctions are essential for rapid nerve conduction and the organization of axonal domains in myelinated axons. Neurofascin155 (Nfasc155) is a glial cell adhesion molecule that is also required for the assembly of these domains. Previous studies have demonstrated that general ablation of Nfasc155 disorganizes these domains, reduces conduction velocity, and disrupts motor behaviors. Multiple sclerosis (MS), a typical disorder of demyelination in the central nervous system, is reported to have autoantibody to Nfasc. However, the impact of focal loss of Nfasc155, which may occur in MS patients, remains unclear. Here, we examined whether restricted focal loss of Nfasc155 affects the electrophysiological properties of the motor system in vivo. Adeno-associated virus type5 (AAV5) harboring EGFP-2A-Cre was injected into the glial-enriched internal capsule of floxed-*Neurofascin* (*Nfasc*^*Flox/Flox*^) mice to focally disrupt paranodal junctions in the cortico-fugal fibers from the motor cortex to the spinal cord. Electromyograms (EMGs) of the triceps brachii muscles in response to electrical stimulation of the motor cortex were successively examined in these awake mice. EMG analysis showed significant delay in the onset and peak latencies after AAV injection compared to control (*Nfasc*^+*/*+^) mice. Moreover, EMG half-widths were increased, and EMG amplitudes were gradually decreased by 13 weeks. Similar EMG changes have been reported in MS patients. These findings provide physiological evidence that motor outputs are obstructed by focal ablation of paranodal junctions in myelinated axons. Our findings may open a new path toward development of a novel biomarker for an early phase of human MS, as Nfasc155 detects microstructural changes in the paranodal junction.

## Introduction

Oligodendrocytes are glial cells that extend myelin membranes around axons in the central nervous system (CNS) [2, 22]. Myelin facilitates saltatory conduction and provides metabolic support to axons [13, 30]. The predominant interaction site between myelin and an axon is formed by the paranodal junction adjacent to the node of Ranvier [33]. Nodes of Ranvier and paranodal junctions flanking the nodes are an important structure supporting saltatory conduction [15]. Rapid transmission of nerve impulses by saltatory conduction is indispensable of normal functions of the nervous system. The paranodal junctions are composed of a 155-kDa isoform of Neurofascin (Nfasc155) on the glial side, and Caspr and Contactin, a glycosyl-phosphatidylinositol (GPI)-anchored neural cell adhesion molecule, on the axonal side [3, 7, 28, 42]. These junctions are thought to have several functional roles, including maintaining action potential propagation [38], restricting nodal proteins for assembly and maintenance of nodes [39], and mediating signal transduction between axons and glia [29]. On the other hand, there is another 186-kDa isoform of Neurofascin, Nfasc186, in neurons. In the absence of Nfas186, sodium channels remain diffusely distributed along the axon [50]. Thus, Nfasc155 and Nfasc186 play distinct and essential roles in the formation of these domains [4, 46]. Glial Nfasc155 is essential for the assembly of the paranodal axoglial junction, while neuronal Nfasc186 is indispensable for stable concentration of sodium channels at the node.

Multiple sclerosis (MS) is the most common demyelinating disease of the CNS, characterized by an initial relapsing–remitting clinical course [12]. In MS, the myelin sheaths are damaged, resulting in significantly delayed conduction velocity and neurological dysfunction [10, 51]. The disruption of paranodal junctions occurs during the early phase of MS [16], and reduced Nfasc155 levels in active MS-lesions have been reported [25]. Moreover, autoantibodies of Nfasc were reported in a subgroup of MS patients, particularly in those with a primary progressive disease course [43]. General ablation of Nfasc155 disorganizes paranodal regions, reduces conduction velocity, and disrupts motor behaviors [50]. However, the impact of focal loss of Nfasc155 remains poorly understood.

In this study, we investigated how the focal loss of Nfasc155 impairs physiological function in vivo. We used the cortico-fugal pathways from the motor cortex to the spinal cord, such as the cortico-spinal, cortico-reticulo-spinal and cortico-rubro-spinal tracts, the major pathways controlling limb movements in rodents [41]. We applied site-specific ablation of Nfasc155 using adeno-associated virus expressing Cre (AAV-Cre), which disrupted the paranodal junctions [34]. Then, the functions of the cortico-fugal pathways were evaluated electrophysiologically by analyzing electromyograms (EMGs) of the forelimb muscles in response to electrical stimulation in the motor cortex in the awake state. We found that focal disruption of paranodal junction impaired conduction through the cortico-fugal pathways from the motor cortex to the spinal cord.

## Materials and methods

### Animals

*Nfasc*^*Flox/Flox*^ mice, in which *Nfasc* exons 2 and 3 were flanked by *loxP* sites, and control wild type (*Nfasc*^+*/*+^) mice were bred for this study and genotyped as described previously [34]. All procedures were approved by the Institutional Animal Care and Use Committee of the National Institutes of Natural Sciences and conducted in accordance with the guidelines of the National Institutes of Health *Guide for the Care and Use of Laboratory Animals.*

### AAV production and purification

**Fig. 1:**
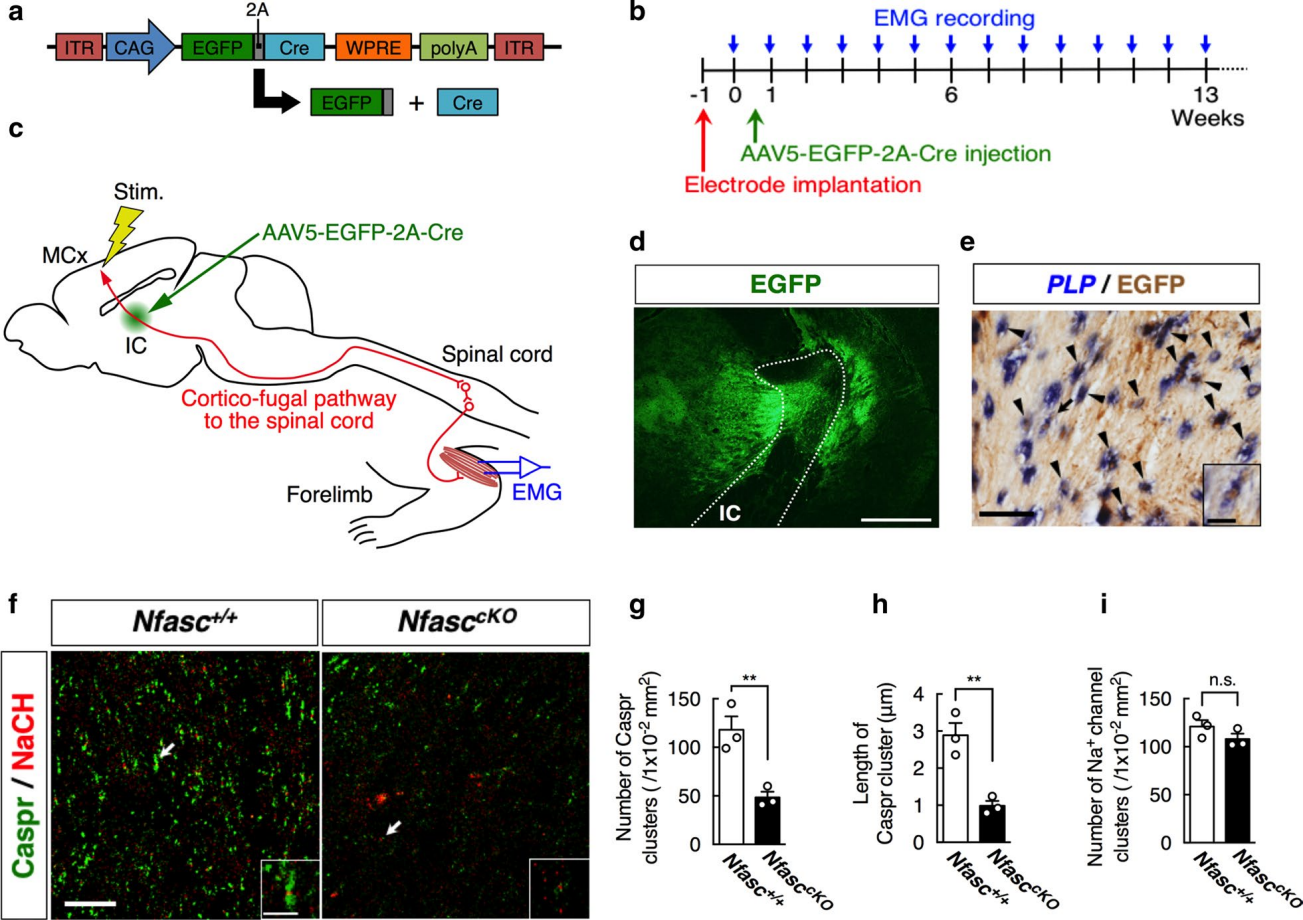
AAV-Cre-induced loss of paranodal junction in the internal capsule. **a** Schematic diagrams showing the design of the AAV-EGFP-2A-Cre constructs. The 2A peptide is cleaved just after translation, and EGFP and Cre recombinase are expressed independently. **b**, **c** Time course and schematic drawing of our experiments. To examine whether site-directed loss of paranodal junctions causes a delay in cortically evoked electromyograms (EMGs), a pair of bipolar stimulating electrodes was chronically implanted into the motor cortex (MCx), and EMG recording electrodes were placed in the triceps brachii muscles contralateral to the cortical stimulation electrodes in 6-week-old *Nfasc*^*cKO*^ and *Nfasc*^+*/*+^ mice. AAV5-EGFP-2A-Cre was injected into the internal capsule (IC) of the mice at week 0. Evoked EMGs by cortical stimulation (Stim.) were recorded every week before (week 0) and after (from week 1 to week 13) AAV injection. **d** Representative micrograph of AAV-mediated EGFP expression (green) in the internal capsule 12 weeks after AAV injection. White dotted lines indicate the boundaries of the internal capsule (IC). Scale bar 250 μm. **e** Doubly staining by in situ hybridization for *PLP* mRNA (blue) and immunostaining with an anti-GFP antibody (brown) in the IC 12 weeks after AAV injection. Arrowheads indicate double positive cells for both *PLP* mRNA and GFP. The inset is a magnified view of the cell indicated by the arrow. Scale bar 50 μm (25 μm in inset). **f** Immunofluorescence staining with an anti-Caspr (green) and anti-Na^+^ channel (red) antibodies in sections containing the IC of *Nfasc*^+*/*+^ (left) or *Nfasc*^*cKO*^ (right) mice 12 weeks after AAV injection. Each inset shows a magnified view of the immunolabeling indicated by the arrow. Scale bar 20 μm (5 μm in inset). Quantification of the number (**g**) and length (**h**) of Caspr-positive paranodes in *Nfasc*^+*/*+^ and *Nfasc*^*cKO*^ mice injected with AAV5-EGFP-2A-Cre vector (Student’s *t* test, number [*t* = 4.623, df = 4, **p < 0.01], length [*t* = 5.319, df = 4, **p < 0.01]; n = 3 mice each). **i** Quantification of the number of Na^+^ channel-positive nodes in *Nfasc*^+*/*+^ and *Nfasc*^*cKO*^ mice injected with AAV5-EGFP-2A-Cre vector (Student’s *t* test, *t* = 1.504, df = 4, *p* = 0.2069; n = 3 mice each). All data are shown as mean ± SEM

EGFP-2A peptide-Cre cDNA was kindly provided by Prof. Akihiro Yamanaka (Nagoya University, Japan) and used as previously described [17]. AAV type 5 harboring CAG promoter-driven EGFP linked to Cre recombinase via a 2A peptide (AAV5-EGFP-2A-Cre, Fig. 1a) was generated and purified as previously described [20, 26, 32]. In brief, HEK293 cells (3 × 10^6^ cells in a 10 cm tissue culture dish) were cotransfected with a pAAV vector plasmid harboring the gene of interest, pAAV-RC2, and pHelper (Cell Biolabs Inc., San Diego, USA). The crude viral lysate was purified with 2 rounds of cesium chloride ultracentrifugation. The titer of the viral stock was determined against plasmid standards by real-time PCR with the following primers: 5′-CCGTTGTCAGGCAACGTG-3′ and 5′-AGCTGACAGGTGGTGGCAAT-3′. The stock was subsequently dissolved in PBS buffer (50 mM HEPES [pH7.4] and 0.15 M NaCl) and stored at − 80 °C.

### AAV injection into the mouse internal capsule for histological examination

To confirm whether AAV5-EGFP-2A-Cre actually disrupted paranodal junctions, the virus was injected into the internal capsule of *Nfasc*^*Flox/Flox*^ (n = 3, all male) and *Nfasc*^+*/*+^ (n = 3, all male) mice. Nfasc155 is expressed in oligodendrocyte of internal capsule and located in the paranodes, while Nfasc186 is expressed in neuron of motor cortex and clustering at the nodes [7]. The AAV5 vector is efficient to drive transgene expression in oligodendrocyte [1]. It also infects axons and is transported mainly anterogradely, not retrogradely to soma [6], and Cre-lox recombination will not occur in neurons. Therefore, *Nfasc155* in glial cells is expected to be specifically ablated, on the other hand, *Nfasc186* in the neuronal side remains intact. Mice were anesthetized with an intraperitoneal injection of ketamine hydrochloride and xylazine hydrochloride (100 mg/kg and 5 mg/kg, respectively) and placed in a stereotaxic frame (Narishige, Tokyo, Japan). After making a skin incision and opening the skull (at 1.0 mm posterior and 2.1 mm lateral to the bregma), 0.3 µl of viral solution (1.0 × 10^9^ viral genome [vg]/ml) was injected slowly (over 3 min) into the internal capsule (at a depth of 2.7 mm) through a pulled glass pipette (outer diameter 50–70 µm) using a syringe pump (UMP3; WPI, Sarasota, USA). The pipette was kept in the same position for 3 min after viral administration then withdrawn. Incisions were closed using wound clips. Animals were sacrificed 12 weeks after virus injection, and injection sites were histologically examined using the methods described below.

### Surgical procedure for electrophysiological experiments

Six-week-old *Nfasc*^*Flox/Flox*^ (n = 7, 6 males and 1 female) and *Nfasc*^+*/*+^ (n = 5, all male) mice were used for electrophysiological experiments (Fig. 1b, c). To painlessly fix the heads of awake mice in the stereotaxic apparatus, the mice first underwent a surgical procedure to attach a prosthesis as described previously [8]. Briefly, each mouse was anesthetized with isoflurane (4%, 100 ml/min with room air) and fixed in the stereotaxic apparatus. The skull was widely exposed and the periosteum and blood on the skull were completely removed. The exposed skull was entirely covered with bone-adhesive resin (Bistite II; Tokuyama Dental, Tokyo, Japan) and acrylic resin (Unifast II; GC, Tokyo, Japan). A small polyacetal U-frame head holder for head fixation was mounted and fixed with acrylic resin on the head of each mouse.

After recovery from the first surgery (3 days later), the mouse was positioned in the stereotaxic apparatus with its head restrained using the U-frame head holder under anesthesia with isoflurane (2%, 100 ml/min with room air). A portion of the skull over one hemisphere was removed to access the motor cortex and internal capsule. A pair of bipolar stimulating electrodes made of 50-μm-diameter Teflon-coated tungsten wires (intertip distance, 300–400 μm) was chronically implanted into the forelimb representation area of the motor cortex, which was identified by intracortical microstimulation (less than 50 μA, 200 μs duration at 333 Hz, 10 pulses). Stimulating electrodes were then fixed therein using acrylic resin. A pair of EMG recording electrodes made of 50-μm-diameter Teflon-coated stranded stainless-steel wires (A-M systems, Carlsberg, USA) were also surgically placed into the triceps brachii muscle of the forelimb contralateral to the side of the cortical stimulation electrodes. The wires were passed subcutaneously and joined to connectors attached to the U-frame.

### Electrophysiological experiments

After full recovery from the second surgery (3 days later), we started recording the evoked EMG responses evoked by cortical stimulation before (week 0) and after (from week 1 to week 13) AAV injection (Fig. 1b). The awake mouse was positioned in a stereotaxic apparatus using the U-frame head holder. Electrical stimulation was applied through the chronically implanted bipolar electrode in the motor cortex (0.6 mA, 100 μs duration, single pulse, 1400 ms interval). The induced EMG signals were amplified (5000×), filtered (15–1000 Hz), rectified by an electrical circuit, stored on a computer at 2 kHz, and averaged for 100 trials.

After the EMG recordings at week 0, AAV5-EGFP-2A-Cre was injected into the internal capsule of *Nfasc*^*Flox/Flox*^ mice using similar methods described above (Fig. 1b, c) under anesthesia with isoflurane (2%, 100 ml/min with room air). EMG recordings of each mouse in awake state were performed once a week on the same day of the week from week 1 through 13 after AAV injection.

### Tissue preparation

Mice were deeply anesthetized with sodium pentobarbital (100 mg/kg, i.p.) and perfused transcardially with 4% paraformaldehyde in 0.1 M phosphate buffer (pH 7.4). The tissues were post-fixed in 4% paraformaldehyde overnight at 4 °C. The post-fixed tissues were cryoprotected overnight in PBS containing 20% sucrose, embedded in OCT compound (Sakura Finetechnical Co., Tokyo, Japan), and cut into 20 µm slices with a cryostat (Leica CM3050, Wetzlar, Germany) for in situ hybridization and immunohistochemistry.

### In situ hybridization

Digoxigenin (DIG)-labeled single stranded riboprobes for *proteolipid protein 1* (*PLP*) [18] were synthesized using T7 RNA polymerase and DIG RNA labeling mix (Roche, Mannheim, Germany). The protocol for in situ hybridization was previously described [21]. Briefly, the sections were treated with proteinase K (40 μg/ml) for 30 min at room temperature and hybridized overnight at 65 °C with DIG-labeled antisense riboprobes in a hybridization solution consisting of 40% formamide, 20 mM Tris–HCl (pH 7.5), 600 mM NaCl, 1 mM EDTA, 10% dextran sulfate, 200 μg/ml yeast tRNA, 1 × Denhardt’s solution, and 0.25% SDS. The sections were washed three times in 1 × SSC (150 mM NaCl and 15 mM sodium citrate) containing 50% formamide at 65 °C, followed by 0.1 M maleic buffer (pH 7.5) containing 0.1% Tween-20 and 0.15 M NaCl. The bound DIG-labeled probe was detected by a 30 min incubation of the sections with anti-DIG antibody conjugated with alkaline phosphatase (Roche), and the color was developed in a solution containing 4-nitro-blue tetrazolium chloride (NBT, Roche) and 5-bromo-4-chloro-3-indolyl phosphate (BCIP, Roche) in the dark at room temperature.

## Immunohistochemistry

Cryosections were immunostained with mouse anti-pan Na^+^ antibody (1:500; S8809, Sigma-Aldrich, St. Lousi, USA), rabbit anti-GFP antibody (1:500; A6455, Life Technologies, Carlsbad, USA), and rabbit anti-Caspr antibody (1:1000; a gift from Dr. Elior Peles, Weizmann Institute of Science, Israel). Sections were irradiated in 10 mM citrate buffer (pH 6.0) for 5 min, heated up to 90 °C in a microwave. After washing with PBS containing 0.1% Triton-X (PBST), sections were blocked with 10% normal goat serum in PBST for 1 h, then incubated with primary antibodies in PBST at 4 °C overnight. After washing with PBST, the sections were incubated with secondary antibodies (1:2000; Alexa488-conjugated goat anti-rabbit IgG and Alexa568-conjugated goat anti-mouse IgG; Molecular Probes, Eugene, USA) for 3 h at room temperature. Sections were mounted and covered with glass coverslips after rinsing with PBST.

Sections used for 3,3′-diaminobenzidine (DAB) staining were blocked with 10% normal goat serum in PBST for 30 min and incubated with rabbit anti-GFP antibody (1:500; Life Technologies) at 4 °C overnight. After washing with PBST, sections were incubated with the secondary antibody (1:400, biotinylated goat anti-rabbit IgG; Vector Laboratories, CA, USA) for 1 h at room temperature, followed by incubation with Avidin/Biotin Complex (ABC) solution (horseradish peroxidase-streptavidin–biotin complex, Vectastain ABC kit; Vector Laboratories) for 1 h at room temperature. The HRP signals were detected by DAB solution with 0.03% H_2_O_2_.

### Data analyses

For histological analysis, the coronal sections of brains from mice injected with AAV vectors were analyzed. Images were acquired by use of Nikon A1R confocal laser scanning microscope and NIS-element software (Nikon, Tokyo, Japan). In the quantification of *PLP*^+^/EGFP^+^ double-positive cells, we considered “double positive cells for both *PLP* mRNA and EGFP” when > 85% of the surface of EGFP-positive cells were wrapped by *PLP*-positive cells, as determined by Z-stack images. In the quantification of paranodes, number of Caspr^+^ clusters per field of view and length of 30–40 Caspr^+^ clusters were quantified in internal capsule. Quantitative analyses were performed by using ImageJ software (NIH).

**Fig. 2:**
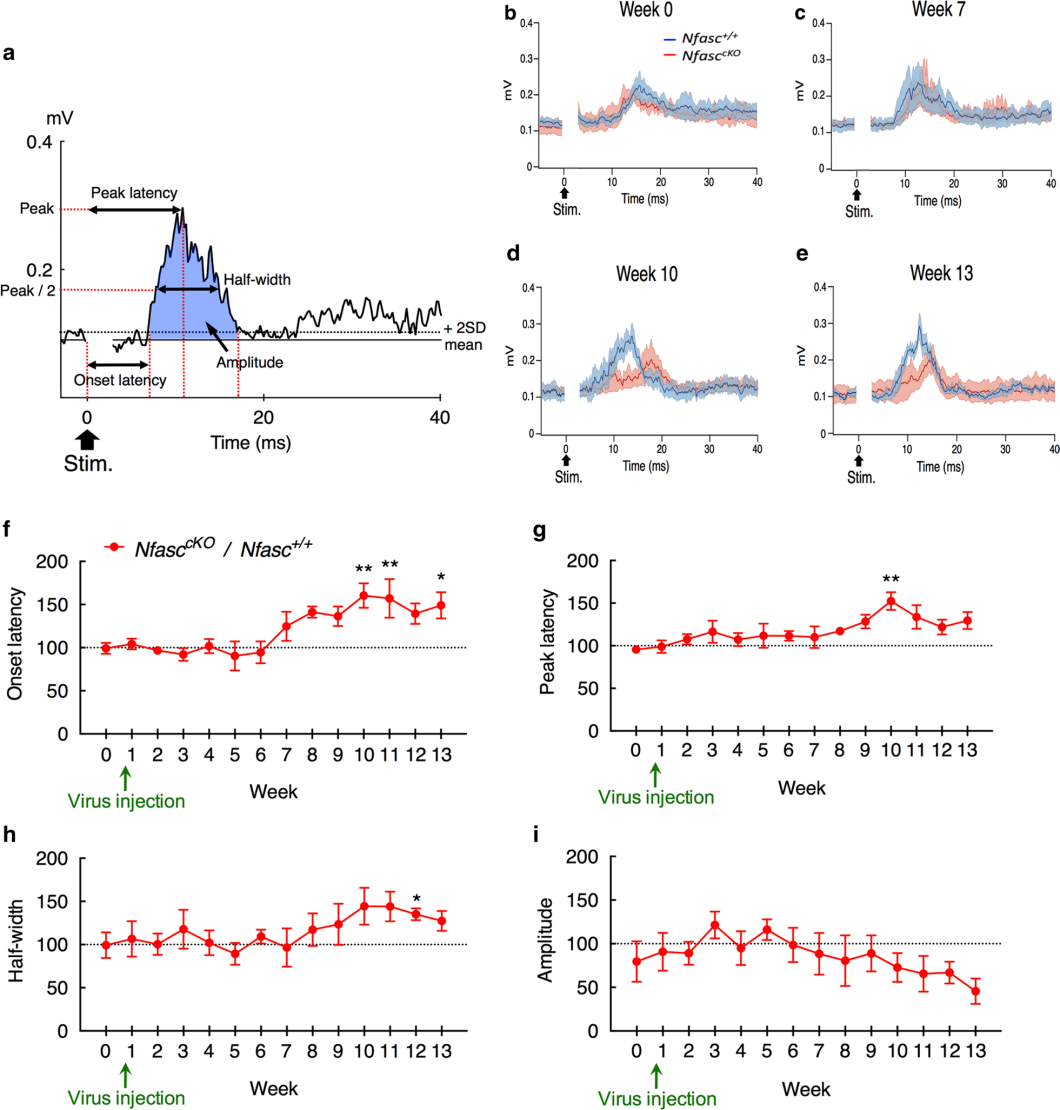
Cortically evoked EMGs in *Nfasc*^*cKO*^ and *Nfasc*^+*/*+^ mice injected with AAV5-EGFP-2A-Cre. **a** Representative EMGs in response to electrical stimulation (0.6 mA, 100 μs duration, single pulse, 1400 ms interval, averaged 100 times) of the motor cortex in *Nfasc*^+*/*+^ mice 12 weeks after AAV injection. Onset latency, peak latency and half-width of the EMGs are indicated. Amplitude of the EMGs is defined as the area over the mean during the response exceeding the level of the mean + 2SD (blue area, see also “Materials and methods”). Cortically evoked population EMGs of *Nfasc*^+*/*+^ (n = 5, blue line) and *Nfasc*^*cKO*^ (n = 7, red line) at week 0 (**b**), 7 (**c**), 10 (**d**) and 13 (**e**). The light-shaded colors represent ± SEM. Ratio (%) of onset latency (**f**), peak latency (**g**), half-width (**h**) and amplitude (**i**) of cortically evoked EMGs in *Nfasc*^*cKO*^ mice compared to those of *Nfasc*^+*/*+^mice before (week 0) and after (from week 1 to week 13) AAV injection (two-way ANOVA followed by Sidak multiple comparison test; onset latency (genotype [F (1,137) = 21.41, **p < 0.01], week [F (13,137) = 2.480, **p < 0.01], genotype × week [F (13,137) = 2.480, **p < 0.01]), peak latency (genotype [F (1,125 = 20.19, **p < 0.01), week [F (13,125) = 1.179, *p* = 0.3024], genotype × week [F (13,125) = 1.179, *p* = 0.3024]], half-width (genotype [F (1,127) = 9.162, **p < 0.01], week [F (13,127) = 0.8992, *p* = 0.5558], genotype × week [F (13,127) = 0.9034, *p* = 0.5515]), amplitude (genotype [F (1,126) = 3.182, *p* = 0.0769], week [F (13,126) = 0.4220, *p* = 0.9595], genotype × week [F (13,126) = 0.4221, *p* = 0.9594])). All data are expressed as mean ± SEM

For electrophysiological analysis, the mean and standard deviation (SD) of the EMG during the control period (0.5-s preceding the onset of the cortical stimulation) were calculated (Fig. 2a). Changes in EMG in response to cortical stimulation were judged to be significant if the EMG exceeded the level of the mean + 2SD. The onset latency of the cortically evoked EMG was defined as the time between the onset of cortical stimulation and the time when the EMG first exceeded this level. The peak latency was defined as the time between the onset of stimulation and the time when the EMG reached its peak. The half-width of the cortically evoked EMG was defined as the width of the EMG at half the height of the peak amplitude. The amplitude of the cortically evoked EMG was quantified as the area of the EMG over the mean when the EMG showed a significant response (blue area in Fig. 2a). Population EMGs were constructed by averaging the EMGs from different animals recorded the same week before and after AAV injection. These parameters of the *Nfasc*^*Flox/Flox*^ mice are expressed as the ratio (%) to those of *Nfasc*^+*/*+^mice.

All statistical analyses were performed using GraphPad Prism 6 Software (GraphPad Software Inc., San Diego, USA). Significance was assessed using Student’s t-test or two-way ANOVA. The Sidak test was used for post-hoc analyses when F ratios were significant. The criterion for a significant difference was *p* < 0.05 for all statistical evaluations. All data are expressed as mean ± SEM.

## Results

### AAV-Cre-mediated ablation of paranodal junction in the internal capsule

AAV-Cre injection into *Nfasc*^*Flox/Flox*^ mice (referred as *Nfasc*^*cKO*^) should result in the deletion of both glial *Nfasc155* and neuronal *Nfasc186* exons 2/3 region [34]. To achieve AAV-Cre mediated specific ablation of *Nfasc155*, we injected AAV5-EGFP-2A-Cre into the *Nfasc*^*cKO*^ mouse internal capsule that composed of glial cells, but not neuronal cell bodies. To confirm that AAV5-EGFP-2A-Cre injection into the internal capsule actually disrupts the paranodal junctions, histological analyses were performed 13 weeks post-injection. Results showed EGFP expression in the internal capsule, indicating the AAV vector was correctly delivered into the targeted region (Fig. 1d). Double-labeling by in situ hybridization for *PLP* mRNA and immunostaining for EGFP showed 89.3 ± 4.5% co-localization of EGFP expression with *PLP*^+^ oligodendrocytes (Fig. 1e). Nfasc155 staining was not successful in the white matter of brain [21, 34, 35, 49], and its loss accompanied disruption of Caspr localization to paranodes in the *Nfasc*^*cKO*^ mice [34]. Therefore, to confirm the loss of paranodal junctions in the internal capsule of *Nfasc*^*cKO*^ mice, immunostaining for Na^+^ channels and Caspr was performed (Fig. 1f). In the *Nfasc*^*cKO*^ mice, both the number and length of Caspr^+^ clusters were significantly reduced compared to the *Nfasc*^+*/*+^ mice (number, **p < 0.01; length, **p < 0.01; Fig. 1g, h). This demonstrates that AAV5-EGFP-2A-Cre injection into the internal capsule of *Nfasc*^*cKO*^ mice efficiently induced the disruption of paranodal junctions. On the other hand, Na^+^ channels remained concentrated probably at nodes (*p* > 0.05; Fig. 1f, i), suggesting minimal effects on Nfasc186. We also found no cortical neurons were labeled with EGFP (data not shown), supporting that Cre-lox recombination did not occur in cortico-fugal neurons.

### Changes in cortically evoked EMGs after ablation of paranodal junction

To investigate whether focal ablation of paranodal junction in the internal capsule affects cortico-fugal nerve conduction, cortically evoked EMGs were recorded for each animal before (week 0) and after vector injection for 13 weeks (once every week during weeks 1–13; Fig. 1b). Typical cortically evoked EMGs recorded from *Nfasc*^+*/*+^ mice 12 weeks after vector injection are shown in Fig. 2a. Figure 2b–e show population EMGs recorded from *Nfasc*^*cKO*^ (red line) and *Nfasc*^+*/*+^ (blue line) mice before (week 0; Fig. 2b) and after (week 7, 10 and 13; Fig. 2c–e) injection of the AAV5-EGFP-2A-Cre vector. At week 0 and 7, cortically evoked EMGs of *Nfasc*^*cKO*^ mice were comparable to those of *Nfasc*^+*/*+^ mice (Fig. 2b, c). However, at week 10 and 13, cortically evoked EMGs of *Nfasc*^*cKO*^ mice, especially during the early phase, were smaller than those of *Nfasc*^+*/*+^ mice. The relative onset and peak latencies of the evoked EMGs (ratio of latency of *Nfasc*^*cKO*^ mice to that of *Nfasc*^+*/*+^ mice) were quantitatively analyzed over time. The onset latencies from week 0 to week 9 did not differ between the two groups (Fig. 2f). In contrast, the onset latencies of the *Nfasc*^*cKO*^ mice were significantly prolonged at weeks 10, 11, and 13 compared to the *Nfasc*^+*/*+^ mice (Fig. 2f; week 10, **p < 0.01; week 11, **p < 0.01; week 13, *p < 0.05; two-way ANOVA followed by Sidak multiple comparison test). Similarly, there were no difference in peak latencies from week 0 to week 9, whereas those of the *Nfasc*^*cKO*^ mice were longer than those of *Nfasc*^+*/*+^ mice at week 10 (Fig. 2g; **p < 0.01). The half-widths and amplitudes of cortically evoked EMGs were also examined. The half-widths of *Nfasc*^*cKO*^ mice were slightly increased after week 8 and significantly longer than those of *Nfasc*^+*/*+^ mice at week 12 (Fig. 2h; *p < 0.05). The amplitudes tended to be gradually decreased after week 7, but there was no significant difference (Fig. 2i). The response of EMGs that we analyzed was not affected by another subcortical pathway because the measuring EMGs of the triceps muscle in response to electrical stimulation of the motor cortex were mediated by the shortest projections from the motor cortex to the spinal cord. Combined, these results demonstrate that the focal loss of paranodal junction in the cortico-fugal pathways slows nerve conduction velocity and increases its dispersion.

## Discussion

In the present study, AAV5-EGFP-2A-Cre was injected into *Nfasc*^*cKO*^ mice, and paranodal junction was disrupted at injected sites in *Nfasc*^*cKO*^ mice. Nfasc155 is expressed in oligodendrocyte at the paranodes, while Nfasc186 is expressed in neuron and clustering at nodes [7]. This AAV vector does not carry the gene to neuronal cell bodies retrogradely [6]. Effects on Nfasc186 were estimated to be minimal because (1) AAV vector does not carry the gene retrogradely to neuronal cell bodies [6], and actually no EGFP labeled cortical neurons were found in this study, and (2) Na^+^ channels at nodes remained intact. Therefore, paranodal junction was specifically disrupted in the *Nfasc*^*cKO*^ mice. In mice with a conventional *Nfasc155* knockout, paranodal junctions are disrupted throughout the CNS but compact myelin is unaffected, resulting in reduced conduction velocity and motor deficits [34, 42]. However, these reports have not addressed whether site-specific ablation of paranodal junction affects the output of the entire pathway, eventually leading to functional abnormalities. In the present study, to determine whether focal loss of paranodal junction in the cortico-fugal pathways affect the electrophysiological properties of the motor system, we analyzed AAV-Cre mediated site-specific ablation of paranodal junction in the cortico-fugal pathways and evaluated EMGs in response to electrical stimulation of the motor cortex. We found that focal disruption of the paranodal junctions in limited regions of the cortico-fugal pathway, such as the cortico-spinal, cortico-rubrospinal and cortico-reticulospinal pathways, influences EMGs evoked by motor cortex stimulation (Fig. 2). Electrophysiological analyses of *Nfasc*^*cKO*^ mice revealed that the onset and peak latencies of cortically evoked EMGs were significantly prolonged (Fig. 2f, g). These defects reflect the slowing of both the fastest and average axonal conduction velocities. The EMG half-widths were elongated, and the EMG amplitudes were slightly reduced (Fig. 2h, i). Abnormal EMG half-widths and amplitudes have also been reported in MS patients [14, 24]. These changes may be caused by conduction blockage in a subset of axons and/or slower conduction velocities in the remaining axons. Furthermore, these findings are consistent with a previous study that the significant changes were observed in later time-points since *Nfasc155* is quite stable once it is incorporated into paranodal junctions in *Nfasc155*^*cKO*^ mice [34].

*Nfasc155* conventional knockout mice did not alter the Na^+^ channel clusters and myelin ultra-structure, but resulted in the accumulation of organelles within the nodes [34]. The disrupted paranodal junction potentially contributes to altered neuronal cytoskeletal organization and axonal trafficking [40], which eventually lead to decreased conduction velocity and amplitudes [9, 34, 47]. Although further studies are needed to confirm whether focal *Nfasc155* loss by AAV injection does not damage the compact myelin, these findings suggest that focal disruption of paranodal junctions influences EMG properties. The currently available data may help to understand the pathology in the early phase of MS.

Infected cells by AAV vectors were also observed outside the internal capsule (Fig. 1d). We measured EMGs of the triceps brachii muscles in response to electrical stimulation of the motor cortex, which were mediated by the shortest (i.e., disynaptic) projections from the motor cortex to the spinal cord, such as the cortico-spinal, cortico-rubro-spinal and cortico-reticulo-spinal tracts [41, 48]. Therefore, infected cells outside the internal capsule had little effects on the evoked EMG.

Disruption of the paranodal junction might impact not only conduction velocity but also motor coordination. Although the disruption of the paranodal junction within a limited region of the cortico-fugal pathways resulted in disturbed conduction along the cortico-fugal pathways, mice showed no apparent motor deficits such as tremor, paralysis, or circling behavior (data not shown). The inconsistency of the impact on motor function may depend on the time of disruption before or after the formation of paranodal junctions. For example, Pillai and colleagues identified hypomotility and severe motor coordination defects in *Nfasc155* conventional knockout mice but not in conditional knockout mice, which show a gradual loss of paranodal junctions after their formation [34]. Another possibility is that the subtle conduction slowing in *Nfasc*^*cKO*^ mice would not be enough to induced obvious motor deficits, while apparent conduction block causes MS symptoms [23].

The pathological study revealed disruption of Nfasc155 localization in the early stage of MS [16] and reduced Nfasc155 levels in active MS-lesions [25]. Interestingly, autoantibodies of Nfasc were reported in a subgroup of MS patients, particularly in those with a primary progressive disease course [43]. Autoantibodies not only to Nfasc but also to other proteins in the paranode, such as Caspr and Contactin, were also reported in chronic inflammatory demyelinating polyneuropathy and Guillain-Barré syndrome [31, 36]. The Nfasc antibodies play, at least, a role in the pathogenesis in MS, because antibody-depleting therapies show potential beneficial responses in MS subgroups with anti-Nfasc antibodies [19].

MS patients frequently display cognitive impairments and psychiatric symptoms [5, 37]. A recent MRI study has demonstrated the relationship between cognitive impairment and white matter lesions in MS patients [27]. Overexpression of PLP, a major component of the myelin sheath, causes overall abnormalities in CNS oligodendrocytes, including abnormal paranodal junctions [18, 45]. A previous study showed that PLP-overexpressing mice display various behavioral abnormalities in relation to cognitive dysfunction [45]. In the CNS, action potentials from different neural circuits are required to reach a target neuron at the appropriate time [44]. If the conduction velocities of various axons are reduced to a certain level, synchronized input might be impaired [11]. Thus, our results further suggest that focal loss of paranodal junctions in one pathway affects not only its own function but also the function of other pathways.

In conclusion, our data revealed that focal loss of paranodal junctions as a result of the loss of Nfasc155 could result in a significant alteration of cortically evoked EMGs in the forelimb muscle. This study will provide us important clues to understand the pathology and early diagnosis of MS.

## Data Availability

The datasets used and/or analyzed in this study are available from the corresponding authors on reasonable request.

## Abbreviations

AAV: Adeno-associated virus
CST: Cerebroside sulfotransferase
CNS: Central nervous system
DTI: Diffusion tensor imaging
EMG: Electromyogram
GPI: Glycosyl-phosphatidylinositol
MRI: Magnetic resonance imaging
MS: Multiple sclerosis
Nfasc: Neurofascin
Nfasc155: Neurofascin155
Nfasc186: Neurofascin186
PLP: Proteolipid protein 1
vg: Viral genome
